# Social Stress-Induced Oxidative DNA Damage Is Related to Prospective Cardiovascular Risk

**DOI:** 10.3390/jcm9113783

**Published:** 2020-11-23

**Authors:** Christiane Waller, Dae-Sup Rhee, Michael Gröger, Manuela Rappel, Tanja Maier, Markus Müller, Edit Rottler, Katharina Nerz, Christopher Nerz, Sebastian Brill, Horst-Peter Becker, Peter Radermacher

**Affiliations:** 1 Klinik für Psychosomatische Medizin und Psychotherapie, Universitätsklinik der Paracelsus Medizinischen Privatuniversität, 90419 Nürnberg, Germany; markus.mueller@klinikum-nuernberg.de; 2 Klinik für Psychosomatische Medizin und Psychotherapie, Universitätsklinikum, 89081 Ulm, Germany; dae-sup.rhee@uni-ulm.de (D.-S.R.); manuela.rappel@uni-ulm.de (M.R.); tanja.maier@uni-ulm.de (T.M.); edit.arbeit@budape.st (E.R.); 3 Institut für Anästhesiologische Pathophysiologie und Verfahrensentwicklung, Universitätsklinikum, 89081 Ulm, Germany; michael.groeger@uni-ulm.de (M.G.); katharina.nerz@tukaneph.de (K.N.); christopher@nerz.me (C.N.); peter.radermacher@uni-ulm.de (P.R.); 4 Klinik für Allgemein-, Viszeral- und Thoraxchirurgie, Bundeswehrkrankenhaus, 89081 Ulm, Germany; sebastian.brill@web.de; 5 Bundeswehrkrankenhaus, 10115 Berlin, Germany; horstpeter.becker@t-online.de

**Keywords:** Trier stress test for groups, PROCAM score, single-cell gel electrophoresis, heart rate variability, noradrenaline

## Abstract

Psychosocial stress increases cardiovascular risk, which coincides with enhanced oxidative DNA damage. Increased sympathetic tone-related catecholamine release causes oxidative stress, which contributes to catecholamine-related cardiotoxicity. Therefore, we tested the hypothesis whether acute psychosocial stress induces oxidative DNA damage, its degree being related to the cardiovascular risk profile and depending on the sympathetic stress response. After assessment of the prospective cardiovascular Münster score (PROCAM) to determine the risk of acute myocardial infarction, 83 male and 12 female healthy volunteers underwent the Trier social stress test for groups (TSST-G). Heart rate variability was quantified by measuring the standard deviation (SDNN) and root mean square of successive differences (RMSSD) between normal-to-normal inter-beat intervals. Salivary α-amylase (sAA) activity was assessed as a surrogate for noradrenaline plasma concentrations. Oxidative DNA damage was determined using whole-blood single-cell gel electrophoresis (“tail moment” in the “comet assay”). A total of 33 subjects presented with a prospective risk of myocardial infarction (risk+) vs. 59 subjects without risk (risk-). The TSST-G stress significantly increased blood pressure, heart rate, and sAA in both groups, while oxidative DNA damage was only increased in the risk+ group. Immediately after the TSST-G, the “tail moment” showed significant inverse linear relations with both SDNN and RMSSD. Acute psychosocial stress may cause oxidative DNA damage, the degree of which is directly related to the individual cardiovascular risk profile and depends on the stress-induced increase in the sympathetic tone.

## 1. Introduction 

Psychosocial stress is associated with cardiovascular risk [1,2], in part due to autonomic dysbalance resulting from a rise in sympathetic tone [3]. Increased superoxide radical formation contributes to catecholamine-related cardiotoxicity [4,5], and enhanced oxidative DNA damage coincides with cardiovascular risk [6]. However, studies investigating the potential impact of social stress on oxidative stress-induced DNA damage are rare [7], and evidence of a putative association with sympathetic stress response is lacking.

Therefore, our study tested the hypothesis that (i) acute psychosocial stress may cause oxidative DNA damage, (ii) the degree of which is directly related to the individual cardiovascular risk profile and (iii) depends on the stress-induced increase in the sympathetic tone.

## 2. Methods

All subjects gave their written informed consent for inclusion before they participated in the study. The study was conducted in accordance with the Declaration of Helsinki, and the protocol was approved by the Ethics Committee of the Ulm University (Antrag Nr. 253/12 - “Auslandseinsatz und Auswirkungen der daraus resultierenden Stresseffekte auf die kardiovaskuläre Gesundheit”, approval date 18 December 2012; trial registration DRKS00022345). We investigated 83 male and 12 female healthy volunteers (mean (range) age: 31 (20–48) years) as a part of the “German Armed Forces Deployment and Stress” study. Three to six soldiers were scheduled to the lab (always at 5:00 p.m.), and a venous catheter was inserted prior to a 30-min resting period. Thereafter, subjects underwent the Trier social stress test for groups (TSST-G), which combines a social stress comprising a 10-min anticipatory stress following a short introduction, a 2-min mock job interview, and a 80-s mental and cognitive arithmetic task [8]. The prospective cardiovascular Münster (PROCAM) score was calculated from eight independent risk variables (age, smoking history, systolic blood pressure, family history of premature myocardial infarction, diabetes mellitus, HDL cholesterol, LDL cholesterol, and triglyceride blood level) to determine the risk of acute myocardial infarction within ten years [9]. Subjects with an at least 1% prospective risk of myocardial infarction were defined as “risk+” vs. subjects without prospective risk (“risk-”). 

**Figure 1:**
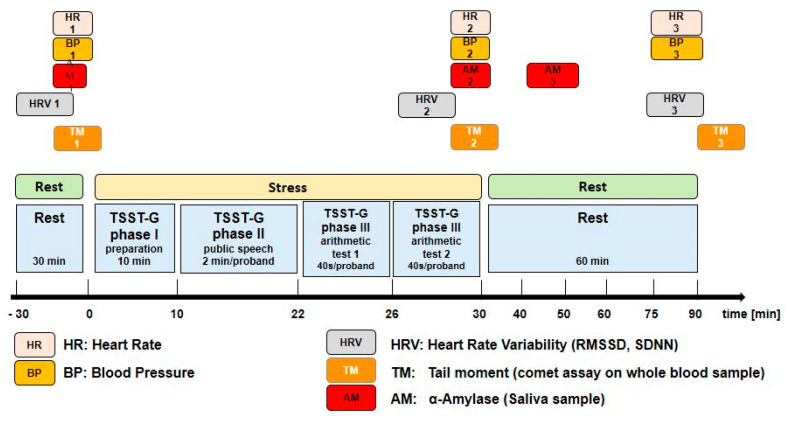
Flow chart of the investigational protocol.

Heart rate variability was derived from 3-lead digital electrocardiogram recordings using the eMotion Faros 180° biosensor (BlindSight GmbH, Schlitz, Germany) with a sampling rate of 500 hertz. Two time domain measures were computed for each subject using 40-s tracings at the stages of TSST-G: standard deviation of all normal-to-normal inter-beat intervals (SDNN), and root mean square of successive differences between normal-to-normal inter-beat intervals (RMSSD) [10,11]. PROCAM scores were lacking in three subjects due to missing smoking information and blood sampling, and in another six due to missing heart rate variability data due to technical problems. Oxidative DNA damage was assessed using single-cell gel electrophoresis of whole blood samples obtained before as well as immediately (within 1 min) and 60 min after the TSST-G. After overnight lysis at 4° C, alkaline (pH = 13, in order to transform alkali-sensitive parts of the DNA into DNA strand breaks) denaturation, electrophoresis performed at 300 mA and 25 V for 25 min, and ethidium bromide staining for the mean “tail moment” (=DNA percentage tail length) were calculated in 100 randomly selected nuclei using image analysis software (COMET Assay IV, vers. 4.3, Perceptive Instruments, Haverhill, UK) [12,13]. Nuclei with a calculated “tail moment” of <0.1 were qualified as “undamaged” [13]. Salivary α-amylase (sAA) activity was measured as a surrogate of plasma noradrenaline concentrations using an enzyme kinetic method as described previously [14]. Samples were obtained before as well as immediately (within 1 min) and 40 min after the TSST-G. Briefly, after dilution, saliva aliquots and standard solutions were incubated with the reagent (a-amylase EPS Sys; Roche Diagnostics, Mannheim, Germany) on a microplate. Thereafter, absorbance was measured at a wavelength of 405 nm using a standard ELISA reader (Anthos Labtech HT2, Anthos, Krefeld, Germany). Due to an insufficient amount of saliva available, data could be obtained in 77 of the participants only. A flow chart of the investigational protocol is shown in Figure 1.

### Statistical Analysis

Data were analyzed with the statistic package SPSS (Ver.25, IBM, USA). Normal data distribution was tested using the Kolmogorov–Smirnov test. Data are presented as median (quartiles, range). Time-dependent within-group effects of the stress exposure on hemodynamic parameters and sAA were analyzed using a Friedmann ANOVA on ranks and a post hoc Mann–Whitney rank sum test with Bonferroni correction for multiple comparisons. Analysis of covariance (ANCOVA) for repeated measures (controlled for gender) was used to assess the group-by-stress effect on the “tail moment” in the “comet assay”. In order to characterize the relationship between the “tail moment” plotted as a function of SDNN and RMSSD, generalized linear modeling with repeated measures and partial correlations was used. Non-linear (i.e., logarithmic, exponential, and/or hyperbolic) models were discarded since the respective calculated regression coefficients did not yield improved mathematical modeling when compared to linear relationships. Significance was stated at *p* < 0.05. 

## 3. Results

**Figure 2:**
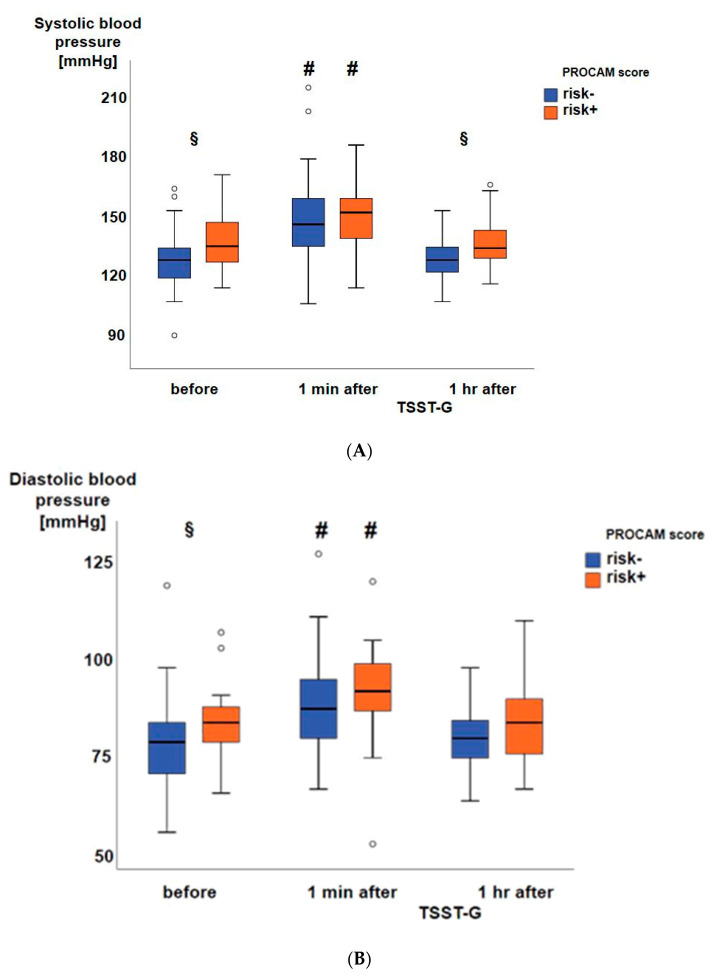

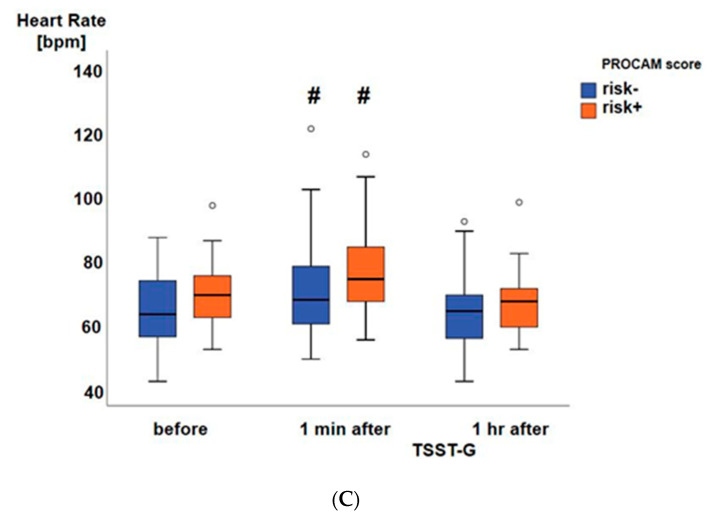
Systolic (**A**) and diastolic (**B**) blood pressure as well as heart rate (**C**) from the 59 subjects without (risk-; blue boxplots) and 33 with (risk+; orange boxplots) cardiovascular risk according to the PROCAM score. Data are presented as median, interquartile range, minimum, and maximum, **#** denotes *p* < 0.05 vs. before and 1 h after the TSST-G, and § denotes *p* < 0.05 between the risk- and risk+ groups.

**Figure 3:**
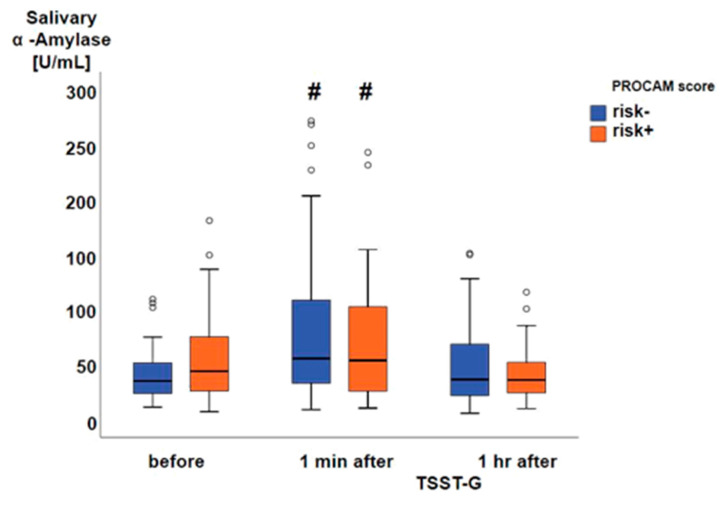
Salivary α-amylase activity from 52 subjects without (risk-; blue boxplots) and 25 with (risk+; orange boxplots) cardiovascular risk according to the PROCAM score. Data are presented as median, interquartile range, minimum, and maximum, and **#** denotes *p* < 0.05 vs. before and 40 min after the TSST-G.

**Figure 4:**
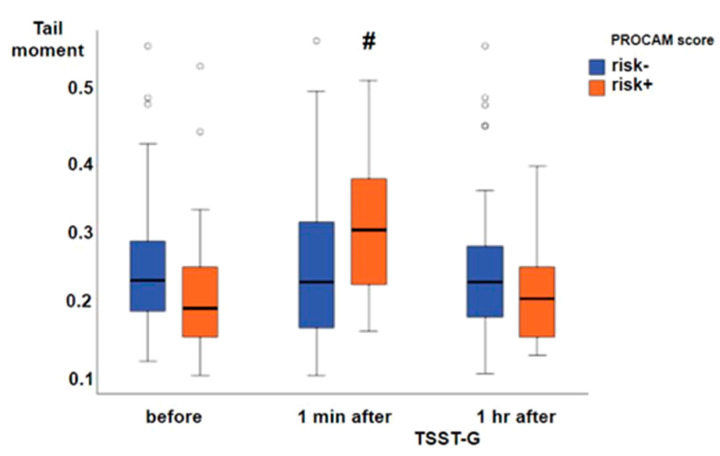
Tail moment in the “comet assay” of whole blood samples from 59 subjects without (risk-; blue boxplots) and 33 with (risk+; orange boxplots) cardiovascular risk according to the PROCAM score. Data are presented as median, interquartile range, minimum, and maximum, and **#** denotes *p* < 0.05 vs. before and 1 h after the TSST-G in the group-by-stress ANCOVA.

**Figure 5:**
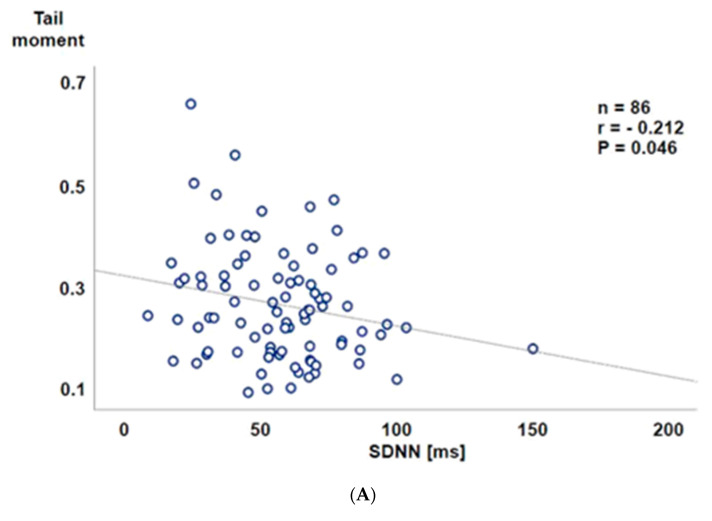

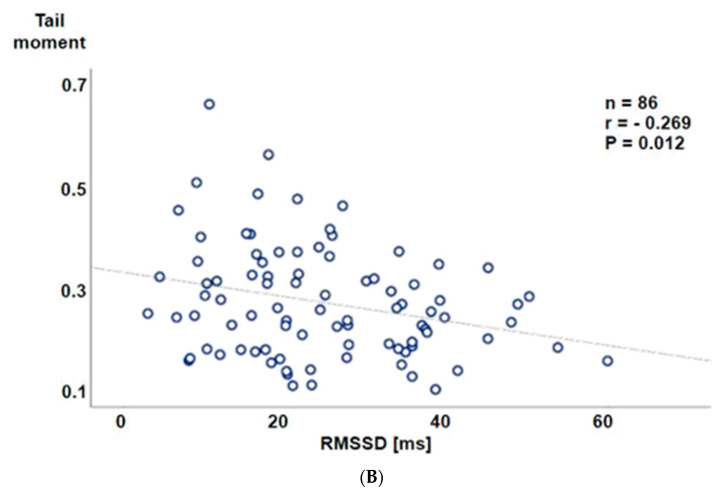
Tail moment in the “comet assay” 1 min after the TSST-G stress plotted as a function of the normal-to-normal inter-beat intervals (SDNN; millisecond (ms)) (**A**, upper panel) and the root mean square of successive differences between normal-to-normal inter-beat intervals (RMSSD; milliseconds (ms)) (**B**, lower panel). Note that the highest immediate post-TSST-G tail moment values were observed with SDNN and RMSSD below the median of the normal values recently reported for healthy volunteers aged 30–39 years, i.e., the mean age of the study population [11,15].

Thirty-three subjects had an at least one percent prospective risk of myocardial infarction (risk+), whereas 59 subjects presented without prospective risk (risk-). Prior to the TSST-G, both systolic (Figure 2A) and diastolic (Figure 2B) blood pressures were significantly higher in the risk+ than in the risk- group, while heart rate (Figure 2C) did not significantly differ between the two groups. One minute after the TSST-G, both systolic and diastolic pressures as well as heart rate were significantly higher than before the TSST-G without any intergroup differences (Figure 2A–C). All hemodynamic values had returned to baseline levels at one hour after the TSST-G, however, the difference between the risk- and risk+ groups present prior to the TSST-G only persisted for systolic blood pressure (Figure 2A). Salivary α-amylase activity was also significantly increased at one minute after the TSST-G, again without difference between the risk- and risk+ groups (Figure 3). TSST-G stress exposure significantly increased oxidative DNA damage in the risk+ group as evidenced by the increased “tail moment” in the “comet assay” demonstrated by the significant group-by-stress effect in the ANCOVA for repeated measures (F(1.85; 164.92) = 5.425, *p* = 0.006). In contrast, no significant effect of the TSST-G could be detected in the risk- subjects (Figure 4). Linear correlations for all participants taken together showed significant inverse relationships of the “tail moment” plotted as a function of SDNN (r = −0.216, *p* = 0.046) (Figure 5A) and RMSSD (r = −0.269, *p* = 0.012) (Figure 5B) immediately after the TSST-G stress.

## 4. Discussion

In the present study, we found that (i) acute psychosocial stress as induced by a TSST-G per se was able to cause oxidative DNA damage, (ii) which was predominant in subjects with increased prospective cardiovascular risk. Furthermore, (iii) there was an inverse relation between the amount of DNA damage and the post-TSST-G parasympathetic activity.

Prolonged psychological stress is associated with increased cardiovascular risk [1,2]. Prolonged psychological stress is also associated with increased oxidative DNA damage as assessed using the “comet assay” [16,17]. Transitory, short-term exposure to psychological stress, e.g., exam periods, may also lead to enhanced oxidative DNA damage [18]. To the best of our knowledge, the present study is the first to demonstrate that even acute psychological stress may exert comparable effects to acute physiological stress, e.g., exercise, in particular beyond the anaerobic threshold [19]. Finally, this finding was only present in study participants with increased prospective cardiovascular risk according to the PROCAM score, which is in good agreement with the more than doubled incidence of oxidative DNA damage in patients with coronary artery disease [6].

Albeit the comet assay was shown to yield robust and comparable results when following thoroughly defined protocols and standardized routine procedures [20], it may still be prone to potential inter- and intra-laboratory variation [21]. In the present study, the baseline tail moment values were comparable to those found in whole blood samples from different groups of slightly younger healthy volunteers using an identical experimental protocol and equipment [12]. Moreover, the median increase in the tail moment was nearly exactly as high as previously observed in healthy volunteers after exposure to hyperbaric oxygen [22], which is well established to induce oxidative DNA damage that can be detected using the comet assay [23].

It could be argued that the Framingham risk score may have been more appropriate for risk estimation than the PROCAM score since it has been referred to as being better at estimating the general cardiovascular risk [24]. However, being based on a localized North American cohort, it was also reported to overestimate the cardiovascular risk in European populations when compared to the PROCAM score [25,26], at least in part due to health inequalities between populations of different ethnicities, socio-economic backgrounds, and family histories [27].

Assessment of heart rate variability, in particular RMSSD, allows determining cardiac vagal tone [28] and, hence, evaluating the relation between sympathetic and parasympathetic activity, i.e., autonomic nervous system balance. It is well established that autonomic dysbalance, which is ultimately associated with reduced heart variability, is directly related to increased cardiovascular risk [29]. In our study, the degree of oxidative DNA damage was inversely related to SDNN and RMSSD, and the highest immediate post-TTST-G tail moment values were observed with an SDNN and RMSSD below the median of the normal values of 37.5/43.4 and 37.7/47.7 (males/females) ms, respectively, as recently reported for healthy volunteers aged 30–39 years, i.e., the mean age of the study population [15]. We did not directly measure catecholamine concentrations, but salivary α-amylase (sAA), which is referred to as a surrogate of plasma noradrenaline concentrations [30], significantly increased at one minute after the TSST-G, however without difference between the risk- and risk+ groups. It should be noted in this context that we may have missed any group effect, because 20% of the sAA data were not available due to the insufficient saliva volume. Furthermore, baseline sAAs were about five to six times lower than in previous investigations [14,30], and the TSST-G exposure increased sAA by about 25 mU/mL only. However, investigating the effect of a TSST-G (rather than a single TSST) in healthy volunteers of a comparable age range as in the present study, other authors reported not only similar baseline sAA values, but also that the TSST-G only moderately increased sAA by about 10 mU/mL [31]. In addition, the direct association between the plasma noradrenaline and the sAA response has been questioned due to variations between types and models of stress as well as the responder patterns [32]. Finally, no matter whether the risk- or risk+ group, the TSST-G was associated with a significant increase in both systolic and diastolic blood pressure one minute after stress exposure, and, moreover, Jarczok et al. reported a significant inverse correlation between overnight urinary norepinephrine and nighttime heart rate variability [33]. Hence, despite the lacking effect on sAA, it is likely that the TSST-G led to increased catecholamine in a particular noradrenaline release: consequently, it is tempting to speculate that the more pronounced oxidative DNA damage indicated by the higher tail moment values in the risk+ group was due to higher TSST-G-induced catecholamine release. This conclusion agrees well with the enhanced superoxide radical formation that contributes to noradrenaline-related toxic effects on cardiomyocytes both in vitro and in vivo [4,5], and the observation that oral antioxidant treatment with vitamin C helps the sympathetic response to exercise by attenuating the increase in noradrenaline blood concentrations in patients after myocardial infarction [34]. In line with this finding, other authors showed that antioxidant pre-treatment also reduced DNA damage after strenuous exercise [18].

### 4.1. Limitations of The Study

Clearly, we did not assess additional parameters of oxidative stress other than the DNA strand breaks using the “comet assay”. However, a recent consensus paper stated that although it “*does not provide insight into the nature of the oxidative modification*”, the comet assay “*is simple and easy to use*” and provides “*general information about strand breaks*” and, thus, is suitable as a general measure of DNA damage [35].

Our study population was part of the “German Armed Forces Deployment and Stress” study. Consequently, the recruitment was unbalanced with respect to distribution between male and female participants. Moreover, the latter were only in the “risk-” group. Nevertheless, we decided to pool the complete dataset: statistical analysis based on the data of the male participants alone yielded the same results (data not shown). Hence, any gender-related effect resulting from a skewed study population can be ruled out.

Our choice of using a 1% threshold for stratifying the study population may represent a study caveat. In fact, usually the PROCAM score differentiates between a low (i.e., <10%), intermediate (i.e., 10–20%), and high (>20%) 10-year risk of coronary events [36]. We chose to use this approach because, due to the participants’ age and their aptitude for foreign deployment, a low overall cardiovascular risk was anticipated in the study cohort. Hence, validation of the findings in an additional study, preferentially in a cohort with at least “intermediate” risk, is warranted. 

Clearly, our data cannot exclude that other effects than the catecholamine-related increase in superoxide radical formation caused the observed aggravation of oxidative DNA damage: using sAA as a surrogate for direct measurement of catecholamine concentrations has inherent weaknesses, and measurement of superoxide radical formation would have required electron spin resonance, which was not available for this study.

### 4.2. Clinical Implications

It is well established that (i) prolonged psychological stress is associated with increased cardiovascular risk [1,2], (ii) oxidative stress plays a critical role in the initiation and progression of atherosclerosis and coronary artery disease (CAD) [37], and (iii) CAD coincides with a two to three times higher incidence of oxidative DNA damage [6]. We now demonstrate that (i) acute psychosocial stress per se may cause oxidative DNA damage, and (ii) the degree of this effect is directly related to the individual cardiovascular risk profile and (iii) depends on the stress-induced increase in the sympathetic tone.
